# Reiterative Mechanisms of Retinoic Acid Signaling during Vertebrate Heart Development

**DOI:** 10.3390/jdb7020011

**Published:** 2019-05-30

**Authors:** Eliyahu Perl, Joshua S. Waxman

**Affiliations:** 1 Molecular and Developmental Biology Graduate Program, University of Cincinnati College of Medicine, Cincinnati, OH 45267, USA; perleu@mail.uc.edu; 2 Medical Scientist Training Program, University of Cincinnati College of Medicine, Cincinnati, OH 45267, USA; 3 The Heart Institute and Division of Molecular Cardiovascular Biology, Cincinnati Children’s Hospital Medical Center, Cincinnati, OH 45229, USA; 4 Division of Developmental Biology, Cincinnati Children’s Hospital Medical Center, Cincinnati, OH 45229, USA; 5 Department of Pediatrics, University of Cincinnati, College of Medicine, Cincinnati, OH 45267, USA

**Keywords:** retinoic acid, heart development, cardiac progenitors, transcriptional regulation, patterning, morphogen, teratogenesis

## Abstract

Tightly-regulated levels of retinoic acid (RA) are critical for promoting normal vertebrate development. The extensive history of research on RA has shown that its proper regulation is essential for cardiac progenitor specification and organogenesis. Here, we discuss the roles of RA signaling and its establishment of networks that drive both early and later steps of normal vertebrate heart development. We focus on studies that highlight the drastic effects alternative levels of RA have on early cardiomyocyte (CM) specification and cardiac chamber morphogenesis, consequences of improper RA synthesis and degradation, and known effectors downstream of RA. We conclude with the implications of these findings to our understanding of cardiac regeneration and the etiologies of congenital heart defects.

## 1. Introduction

The vertebrate heart is the first organ to form and function in all vertebrates. Its ability to function properly during embryogenesis and postnatal life is vital for both the normal development of other organs and an embryo’s long-term survival. We now recognize that vertebrate heart development during embryogenesis is a progressive process that integrates many intricate cellular interactions. Consequently, given the complexity of heart development, it is not surprising that congenital heart malformations are the most common human birth defects, accounting for nearly one-third of all major congenital anomalies and an incidence of ~1% of all live births [1]. The congenital heart diseases (CHDs) that arise from these malformations can be due to genetic or environmental causes [2]. Hence, understanding the mechanisms that promote normal and aberrant vertebrate heart development is critical for generating preventative and therapeutic strategies for the broad etiologies of CHDs.

**Figure 1:**
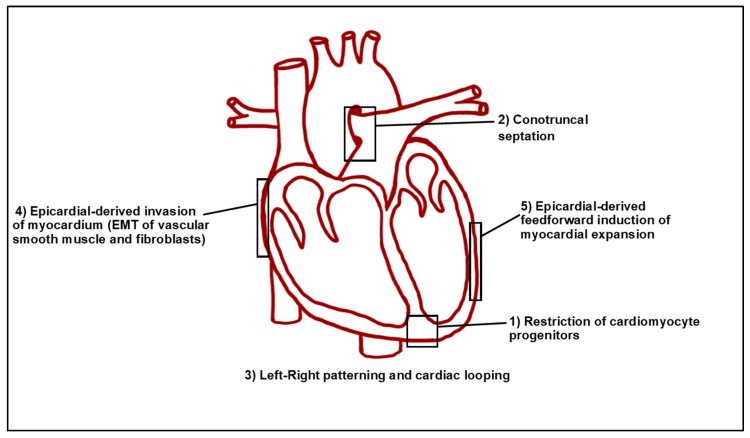
Tightly controlled levels of RA signaling regulate multiple aspects of vertebrate heart development. Schematic of a four-chamber mammalian heart. **1**) RA restricts the number of CM progenitors during early development within the anterior lateral plate mesoderm (ALPM). **2**) Multiple levels of RA transport, synthesis, and signaling regulate proper conotruncal septation and the formation of the OFT. **3**) During the broader processes of cardiac morphogenesis, RA receptors (RARs) have been shown to be crucial for appropriate left-right patterning, cardiac looping, and development of the inflow tract. **4**) RA is necessary for the proper invasion of epicardial-derived cells into the ventricular myocardium, their subsequent differentiation into fibroblasts, and for the regulation of proper vascular smooth muscle and coronary vessel formation. **5**) RA also promotes CM proliferation via a feedforward loop that drives its release from the epicardium and is necessary in the epicardium for regeneration.

Retinoic acid (RA) is the most active metabolic product of Vitamin A (retinol) within vertebrate embryos. Although RA signaling is necessary for normal vertebrate heart development, it is critical that the levels of embryonic RA are maintained within an appropriate range as both decreases and increases in RA signaling can result in congenital heart malformations [3,4]. Indeed, at least in animal models, Vitamin A deficiency (VAD) can result in a variety of severe congenital heart abnormalities, including the absence of the coronary vascular network and a ballooned, non-compartmentalized, randomly-positioned heart with inflow and outflow tract anomalies [5,6,7,8]. In humans, although there is less incidence of VAD in Western societies [9], it is still pandemic and affects ~15% of pregnant women worldwide [10]. Excessive intake of RA or Vitamin A (called hypervitaminosis A) is highly teratogenic in humans and can result in transposition of the great vessels (TGV), tetralogy of Fallot (TOF), double-outlet right ventricle (DORV), persistent truncus arteriosus (PTA), atrioventricular septal defects (AVSDs), and aortic-arch artery malformations [11,12]. Significantly, RA is used therapeutically, such as for severe cases of nodular acne vulgaris. In the US, women of childbearing age are required to be on two forms of contraceptive when being treated with isotretinoin (an isoform of RA) for acne, indicative of RA’s significant teratogenicity [13]. Furthermore, due to the emphasis of dietary vitamin supplementation, it is estimated that 75% of people in developed nations regularly ingest more than the recommended daily allowance for Vitamin A [14]. Despite the attention that RA signaling has received over the past 70 years with respect to heart development, there is still a considerable way to go until we have a complete understanding of the precise mechanisms by which RA signaling reiteratively directs the multitude of processes required for normal heart development (Figure 1). Here, we highlight known roles of RA signaling during early vertebrate heart development, the current understanding of the requirements of RA signaling during CM specification, and the implication that downstream responses to RA signaling contribute to specific heart malformations.

## 2. Initial Insights into the Roles of RA Signaling during Heart Development

**Figure 2:**
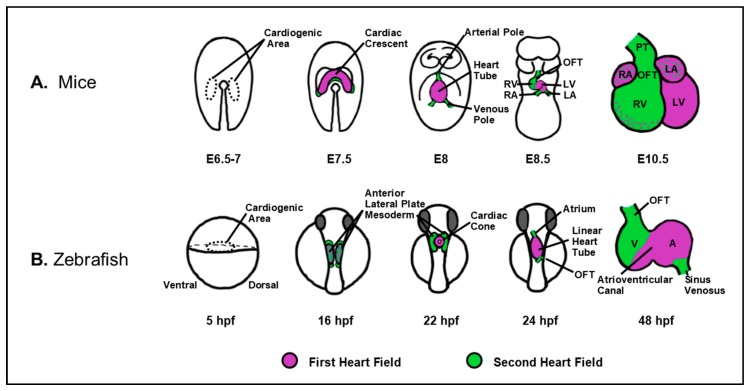
Vertebrate cardiac development proceeds via the contribution of waves of differentiating progenitors, as depicted in mice (**A**) and zebrafish (**B**). During early heart development, cardiac progenitors are specified bilaterally within the lateral plate mesoderm and subsequently coalesce as the cardiac crescent in mice and cardiac cone, in zebrafish. In mice and zebrafish embryos, the FHF (magenta) first forms the cardiac tube followed by contribution of the SHF (green) to the poles of the developing heart. Subsequently, the heart undergoes an expansion process, including ballooning of the cardiac chambers, looping, and the generation of the septa in the case of a four-chamber heart. A: atrium; E: embryonic day; HPF: hours post-fertilization; LA: left atrium; LV: left ventricle; OFT: outflow tract; PT: pulmonary trunk; RA: right atrium; RV: right ventricle; V: ventricle.

The study of RA signaling has a rich history, initiating with the investigation of VAD in the 1920s [15,16]. Collectively, a series of studies led by Dr. Josef Warkany were the first to suggest that the levels of Vitamin A, and consequently RA, need to be tightly-regulated for the establishment of normal vertebrate heart development. In the late 1940s and early 1950s, Wilson and Warkany showed that rat embryos harvested from mothers fed a VAD diet exhibited outflow tract (OFT) and aortic arch defects [5,6,17,18]. At about the same time, Cohlan first demonstrated that excessive intake of Vitamin A (hypervitaminosis A) also causes a spectrum of congenital anomalies in rat embryos [19]. Subsequently, Warkany et al. showed that hypervitaminosis A caused OFT and septal defects in mice [20]. As indicated above, we now understand that excessive RA itself is a potent teratogen, which can occur due to hyper-supplementation, excess liver intake, or oral RA therapy, and may result in RA embryopathy and CHDs that include a spectrum of OFT and septal defects [8,9,11,12,21,22,23,24]. Since these initial experiments, studies in a variety of vertebrate animal models have demonstrated that inappropriate RA signaling results in a similar spectrum of congenital malformations [7,25,26,27,28,29,30,31,32,33,34,35,36]. Importantly, our understanding of the role(s) of RA signaling during heart development have evolved considerably, in part due to the greater realization that vertebrate heart development is a dynamic process [21,37,38,39,40,41,42,43,44,45]. We now know that the differentiation of cardiac progenitors occurs continuously, but functionally and genetically in two waves [4,39,46,47,48,49,50] (Figure 2). In all vertebrates, cardiac progenitors are specified bilaterally at positions within the anterior lateral plate mesoderm (ALPM). Subsequently, progenitor cells move toward the midline to fuse, forming the rudimentary heart tube. The first heart field (FHF), the earlier differentiating wave of cardiac progenitors, forms the primitive heart tube. The second heart field (SHF), which comprises later differentiating cardiac progenitors in the pharyngeal mesoderm adjacent to the FHF, then accrete to both poles of the developing heart. In the more complex four-chambered hearts found in birds and mammals, the left ventricle is predominantly derived from the FHF, while the SHF contributes to both atria, the right ventricle, and the outflow tract (Figure 2A). In the two-chambered heart of zebrafish, the SHF gives rise to almost half the ventricle, the bulbous arteriosus in the outflow tract, and a small portion of the atrium [39,46,47] (Figure 2B). 

## 3. RA Signal Restricts Cardiac Progenitor Specification

### 3.1. Restriction of Cardiac Progenitors Fields within the ALPM

Proper levels of RA signaling are required reiteratively throughout vertebrate heart development, with defined roles during early patterning of the cardiac progenitor fields, OFT development, epicardial cell determination, and cardiac regeneration (Figure 1). RA signaling’s requirement in early cardiac progenitor patterning has received a significant amount of attention. Because RA signaling is both necessary and sufficient to promote posterior fates along the anterior–posterior (A-P) axis early in vertebrate embryos [51,52], it is logical that its earliest requirements in cardiogenesis reflect this role. Indeed, the major RA producing enzyme, Aldehyde dehydrogenase 1 family member A2 (Aldh1a2—formerly called Raldh2) is expressed within the ALPM and somites that are immediately posterior to the cardiogenic field [53,54,55]. As discussed in a number of informative reviews in greater detail [56,57], it was first hypothesized that RA signaling partitions naïve cardiac progenitors into atrial and ventricular CMs by promoting atrial identity at the expense of ventricular identity. This hypothesis, attractive due to its simplicity, was derived from studies in zebrafish, chicken, and mouse embryos that showed overtly differential sensitivity of the atrial and ventricular chambers to modulation of RA signaling [29,30,58,59]. However, these studies were largely based on morphological analysis of the hearts. As argued below, the use of cardiac progenitor markers for the FHF and SHF, updated fate maps, and the quantification of cardiomyocytes within the cardiac chambers of these models have challenged this hypothesis. Our position is that RA signaling does not strictly promote atrial identity within CMs in vivo within the vertebrate embryo. Despite the lack of in vivo support for a role in determining atrial and ventricular identity, this does not diminish the observations that certain RA concentrations can be used to preferentially induce atrial identity in embryonic and induced pluripotent stem cells [60,61,62,63,64]. Thus, manipulating RA signaling levels can be employed in directed differentiation protocols to enhance the generation of specific CM populations in in vitro contexts, which clearly is valuable for tissue engineering and regenerative approaches that may be used to confront cardiac diseases.

**Figure 3:**
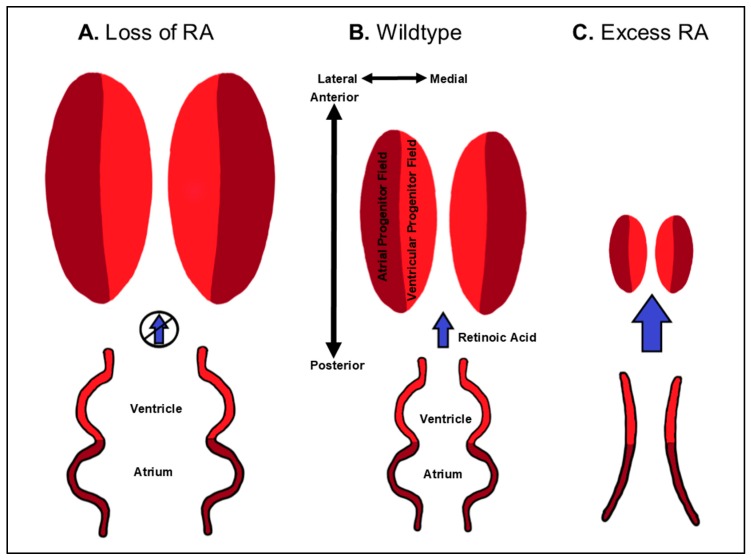
Schematic depicting restriction of CM specification by RA signaling. Levels of RA are indicated by blue arrows. Loss of RA results in a marked expansion of both chambers (**A**) compared to wildtype hearts (**B**). High doses of RA can inhibit the specification of both ventricular and atrial CMs (**C**), even completely eliminating cardiac specification with the highest dosages. While extremes of RA signaling perturbation are depicted here, progressive increases in RA signaling can cause differential effects on ventricular and atrial specification. The schematic depicted is primarily derived from studies in zebrafish.

The prevailing hypothesis for the early requirement of RA signaling in vivo is that it restricts the cardiac progenitor fields within the ALPM (Figure 3 and Table 1), again as part of its role in A-P patterning along the embryonic axis. This hypothesis originated from studies in zebrafish. It was demonstrated that both zebrafish mutants bearing *aldh1a2* mutations, called *neckless* (*nls*), and inhibition of RA signaling in embryos using pharmacological antagonists result in increased specification of cardiac progenitors within the ALPM, producing larger hearts with increased number of atrial and ventricular CMs [41,65]. Subsequently, studies in the frog *Xenopus laevis* and mice also indicated that RA signaling limits cardiac specification within the ALPM [66,67]. In mice, *Aldh1a2* mutants have a posterior expansion of cardiac progenitor marker expression [67,68]. Despite the expansion, it is still unclear if RA signaling strictly restricts SHF specification or aspects of both FHF and SHF specification. It has been suggested that RA signaling restricts the SHF in mice [67]. However, in the absence of RA signaling, the SHF progenitor cells fail to differentiate in mouse embryos, which accounts for the observed OFT defects. In zebrafish, whether RA signaling differentially affects FHF or SHF progenitors has not been reported. Nevertheless, consistent with the observation that RA signaling restricts both ventricular and atrial progenitors, caged-fluorescein-mediated lineage tracing in zebrafish has shown that the ventricular and atrial progenitors lie in a medio-lateral orientation within the ALPM [46,69,70] (Figure 3). It had been proposed that ventricular and atrial progenitors have an A-P relationship within vertebrate embryos [53], which at one point was argued to support the RA-chamber patterning hypothesis mentioned above. However, the most recent fate-maps of the ALPM in chick embryos have suggested that the orientation of ventricular and atrial progenitors is also medio-lateral [71]. Thus, current data support that RA signaling has a conserved requirement restricting the cardiac progenitor fields within the ALPM of vertebrates.

### 3.2. Coordination of Adjacent Cardiac and Forelimb Progenitor Fields by RA

RA signaling patterns cell fates in all three embryonic germ layers along the A-P axis [38,89]. Thus, the expansion of the cardiac progenitor field within the ALPM due to insufficient RA signaling indicates that this consequence might affect neighboring progenitors. In addition to cardiac defects, *Aldh1a2* mutant mice and zebrafish embryos lack forelimbs (the pectoral fins in zebrafish) [41,90]. Consistent with this hypothesis, studies in zebrafish and mice have shown that loss of RA signaling inversely affects fate decisions between the adjacent cardiac and forelimb progenitors fields within the ALPM [41,90,91,92]. Caged-fluorescein lineage tracing has shown that in RA signaling-deficient zebrafish embryos, cardiac progenitors arise from a region of the posterior ALPM that normally harbors forelimb progenitors [41]. Furthermore, in zebrafish *tbx5a* expression, the ortholog of *TBX5* in humans that commonly results in Holt-Oram (hand-heart) syndrome and is essential for venous pole development [93,94], normally marks a continuous band of cells that encompasses cardiac and forelimb progenitors [95]. This continuous band of expression within the ALPM subsequently separates into anterior cardiac and posterior forelimb mesenchyme populations as development proceeds. However, in RA signaling-deficient zebrafish embryos, all the *tbx5a*^+^ cells contribute to the heart field, consistent with a posterior expansion and partitioning of fates within this expression field [95]. Furthermore, there is mechanistic evidence that the coordination of this fate decision occurs through RA’s repression of FGF signaling, which promotes cardiac specification. In *aldh1a2/nls* zebrafish and *Aldh1a2* mouse mutants, the expression of *Fgf8* homologs is expanded in cardiac progenitors [67,68,96,97,98]. In zebrafish, inhibition of Fgf8a is sufficient to restore pectoral fin development in RA signaling-deficient embryos. Thus, within vertebrate embryos, RA signaling has a conserved requirement limiting FGF signaling within the ALPM, which is necessary to coordinate proper heart and forelimb development [90,92].

### 3.3. Dose-Dependent Differential Regulation of Atrial and Ventricular Progenitor Specification by RA

The teratogenicity of RA demonstrates the critical need to limit RA signaling within embryos. With respect to early patterning, increases in RA signaling support the hypothesis that RA restricts cardiac specification more generally (Figure 3). Specifically, treatment of zebrafish and chick embryos and embryonic stem cells with high concentrations of RA can eliminate CM specification [30,33,58,99,100,101]. Thus, loss and gain of RA signaling have conserved requirements limiting ventricular and atrial CM specification. Despite the ability of high levels of RA signaling to eliminate CM progenitors, the effects of intermediate increases in RA signaling are rather nuanced, which can in part explain the interpretation that RA signaling promotes atrial identity at the expense of ventricular identity. Interestingly, depending on the level of excess RA signaling, dramatically different and seemingly paradoxical effects on ventricular and atrial specification are observed, which are demonstrably not the clear converse of loss of RA signaling. For instance, treatment of zebrafish embryos with persistent, yet very low concentrations of RA can promote both ventricular and atrial specification [34]. Yet, treatment of zebrafish embryos with varying levels of intermediate concentrations can independently affect ventricular and atrial specification, which parallels the degree of induced posteriorization along the A-P axis across the three germ layers. Lower-intermediate concentrations of RA can promote atrial specification without affecting ventricular progenitor specification, while higher-intermediate concentrations start to repress ventricular specification without affecting atrial specification. As the previously used RA concentrations in models like chickens and zebrafish did not predominantly eliminate cardiac progenitors [29,30], it is clear how the overt morphology of these hearts could lead to the interpretation that RA signaling promotes atrial identity at the expense of ventricular identity. Importantly, in zebrafish, cardiac defects due to loss of *cyp26a1* and *cyp26c1*, which are necessary to degrade RA, recapitulate the defects observed with lower-intermediate RA treatments, reflecting the lower levels of endogenous retinoids within these embryos compared to exogenous treatments [42,44,83,102] (Table 1). Since it is established that RA signaling acts as a morphogen within the zebrafish embryo [40,103,104,105,106], we have proposed that this concentration-dependent effect of RA on cardiac specification is due to a flattening of the RA signaling gradient [3]. Similar to what is observed with loss of RA, increased RA signaling affects fate decisions between cardiac and adjacent progenitors within the ALPM. Lineage-tracing in embryos deficient for both Cyp26a1 and Cyp26c1 in zebrafish embryos have shown that the resulting increase in RA signaling promotes an anterior shift in the cardiac field. This expansion of atrial progenitors with moderate increases in RA levels is at the expense of more anterior cranial vasculature progenitors [44]. Thus, like loss of RA signaling, increases in RA signaling promote fate decisions between cardiac and adjacent progenitor fields within the embryo.

## 4. Consequences of Perturbing Components of the RA Signaling Pathway during Heart Development

### 4.1. Congenital Heart Malformations from Loss of RA Synthesis

RA is generated through a series of enzymatic steps from Vitamin A [3,4]. As indicated in part above, the importance of proper RA signaling to vertebrate heart development has been revealed through studying mutants involved in the production and degradation of RA, as well as in the storage of its precursors. In humans, mutations that affect the gene *Stimulated by retinoic acid 6* (*STRA6*), which is required for the translocation of retinol into cells, are associated with Matthew Wood Syndrome, a disease characterized by dextrocardia, OFT, and septal defects [75,76,77,88] (Table 1). However, murine KOs for *Stra6*, as well as enzymes involved in the transport of retinol (Retinol binding protein 4 (Rbp4)) and storage of retinol (Lecithin retinol acyltransferase (Lrat)), do not have overt cardiac defects themselves [24,107,108,109,110,111]. Therefore, we will not focus on them here, but refer to other reviews covering their requirements in more detail [3,112]. In contrast to loss of enzymes involved in the transport and storage of retinol, perturbation of the enzymes required to synthesize RA produce dramatic cardiac defects. A long-standing view was that the local production of RA through tissue-specific expression of Aldh1a2 produced the embryonic RA gradient. However, a mutagenesis screen in mice first showed that Retinol dehydrogenase 10 (Rdh10), which is required for the conversion from retinol to retinal also has localized expression that helps generate the RA gradient [85]. Indeed, with respect to the heart, the most severe defects in *Rdh10* mutant mice appear to phenocopy *Aldh1a2* KO mice as both exhibit linearized, dilated, and unlooped hearts [28,67,85] (Table 1). However, the severity of the overall phenotype of *Rdh10* KO appears to vary with genetic background [86,87,113]. Although a *rdh10a* mutant zebrafish has yet to be reported, we have found that Rdh10a-depleted zebrafish embryos have enlarged hearts with increased CM number, similar to *aldh1a2* zebrafish mutants [114]. As described above, in both *aldh1a2/nls* mutant zebrafish and *Aldh1a2* KO mice, there is a posterior expansion of cardiac progenitors [41,65,68,115]. Recent work has shown that RA signaling within the posterior SHF regulates interactions between T-box transcript factors to promote proper heart development. The transcription factor Tbx1, which is associated with DiGeorge syndrome (DGS) in humans [72,73], is a conserved regulator of SHF accretion to both cardiac poles of the heart [116,117]. During the early stages of heart tube elongation in mice, a subset of *Tbx1*-expressing cells in the posterior SHF downregulates the arterial pole progenitor program via the expression of *Tbx5* at the future venous pole [43]. The posterior *Tbx5* activation in this population requires RA for its expression, as blocking the RA signaling that promotes the caudal *Tbx5* expression leads to AVSDs [43]. While *Aldh1a2* KO mice have a posterior expansion of the cardiac progenitor field and OFT defects due to a failure of the SHF to differentiate, pleotropic effects on other tissues that contribute to the heart also underlie these defects. Specifically, *Aldh1a2* KO mice have improperly localized cardiac neural crest within the OFT [118]. Thus, in addition to proper patterning of cardiac progenitors along the A-P axis, the production of RA is necessary to partition the SHF and for the proper incorporation of neural crest into the OFT.

### 4.2. Congenital Heart Malformations from the Inability to Limit RA

CYP26A1 is the major RA-degrading enzyme during early vertebrate development [80,81,82,119]. In humans, loss of *CYP26A1* expression is indirectly associated with DGS, which results in OFT and aortic arch defects. DGS is predominantly associated with heterozygous deletion of the 22q11.2 region on chromosome 22. The defects found with DGS are largely attributed to loss of the *TBX1* gene [120]. Numerous studies have pointed to a mutually repressive signaling loop between Tbx1 and RA signaling during pharyngeal arch artery development, in part through Tbx1 promoting Cyp26a1 expression [72,115,121,122]. In *LgDel/+* mice, a model for DGS that are hemizygous for 24 different genes, pharyngeal defects are enhanced with RA treatment, supporting the idea that there is increased sensitivity to RA in this model [123]. Likewise, the spectrum of congenital malformations in murine and zebrafish *Cyp26a1* mutants is consistent with increased RA levels [80,82,83,102,124,125]. In mice, *Cyp26a1* KO results in looping defects [80]. Although *Cyp26c1* KOs alone do not have overt cardiac defects [83], compound *Cyp26a1* and *Cyp26c1* KOs produce a more severe phenotype, indicating that there is some functional redundancy between these enzymes within embryos [83]. As already discussed above, the earliest cardiac defect observed in Cyp26-deficient zebrafish embryos is an increase in atrial specification at the expense of adjacent anterior endothelial progenitors, due to an anterior shift in the cardiac progenitor field residing in the ALPM [44]. However, we found that modest depletion of the Cyp26 enzymes can produce OFT defects independent of the early patterning defects. Remarkably, the cardiac defects in these Cyp26-depleted embryos results from two mechanisms: First, there is a failure of SHF progenitors to join the OFT. Instead, they contribute to the pharyngeal arch arteries; Second, FHF ventricular CMs are extruded from the heart tube, due to disruption of their cell polarity and the surrounding extracellular matrix [42]. While Cyp26 enzymes facilitate the degradation of RA, dehydrogenase/reductase 3 (Dhrs3) is necessary to limit available embryonic RA levels, as the major embryonic reductase responsible for converting retinal back into retinol for storage. *Dhrs3* null mice and Dhrs3a-deficient zebrafish have increases in RA signaling, consistent with its role in limiting RA production [26,74,126,127]. Importantly, *Dhrs3* KO mice exhibit a spectrum of congenital heart defects, including OFT and septal defects [74], as well as aberrant invasion and migration of the epicardial cells into the myocardium, which results in thinning of the myocardium and aberrant coronary vessel formation [26] (Table 1). Thus, cardiac integrity within the nascent heart tube, OFT, and epicardial development are particular sensitive to increased levels of RA. 

### 4.3. Congenital Malformations from Loss of Retinoic Acid Receptors

RA is a ligand for cognate nuclear hormone receptors that produce direct transcriptional output [128]. Canonical RA signaling is integrated through RA receptors (RARs) that bind RA response elements (RAREs) upon heterodimerization with retinoid X receptors (RXRs) [103]. In mammals, there are 3 RARs (-α, -β, and -γ), each with numerous RAR isoforms. There appears to be significant functional redundancy between them, given that mutation of a single isoform does not result in significant overt cardiac defects. Double KOs of *RARα* with *RARβ*, *RARγ*, or *RXRα* exhibit a range of OFT defects (Table 1), including DORV, PTA, AVSDs, and aortic arch defects [35,78,79,129]. However, studies of hearts in *RXRα* KO mice have indicated that KO of this single RXR isoform have subtle cardiac defects due to premature differentiation of ventricular CMs. Although this phenotype can be partially recapitulated in *RARα* KO and VAD mice [130,131,132,133,134,135], supporting that it may be mediated by RA signaling, a caveat is that RXRs can dimerize with other nuclear hormone receptors [128]. Nevertheless, work specifically examining the OFT defects of *RARα1*; *RARβ* double mutants has shown that the SHF fails to differentiate in these mutants [79]. Furthermore, these mutant mice fail to express the transcription factor Gata4, a marker of cardiac progenitor specification, in the caudal portion of the SHF. Importantly, both *RARα1*; *RARβ* mutants and *Gata4^+/−^*; *Gata6^+/−^* heterozygous mice are born with a common arterial trunk, supporting a relationship between RA signaling and Gata factors during OFT development [79,136]. 

In addition to the apparent genetic interaction between RA signaling and Gata4, it appears that the OFT septation defects observed in *RARα1*; *RARβ* double mutant mice may be due to a direct downstream response of RARs within the mesodermally-derived OFT to limit TGF-β signaling [79]. Interestingly, ectopic expression of RA signaling causes OFT/cushion defects that occur via the direct repression of *Tbx2*—and consequently TFG-β—lending support to a broader antagonistic relationship between RA and TFG-β signaling that needs to be balanced for proper vertebrate OFT development [137]. In contrast to murine models, it has been proposed that RARs have distinct and non-redundant roles in other vertebrates. For instance, in chick embryos, it has been shown that RARγ regulates left–right patterning and looping during cardiac morphogenesis, and that RARα2 is required for the formation of the inflow tract [84]. Due to the genome-wide duplication in teleosts, zebrafish have 4 RARs (-αa, -αb, -γa, and -γb, yet they lack β genes) and 6 RXRs (-αa, -αb, -βa, -βb, -γa, and -γb) [138,139]. There is significant redundancy of RARs in zebrafish [140]. However, we found that depletion of the zebrafish RARαb1 isoform results in slight cardiomegaly due to increased CM specification [34]. Surprisingly, these defects appear to be due to inappropriate feedback that results in a gain of low levels of RA signaling. Collectively, these data suggest that RA and its various receptors intimately interact with other signaling networks and likely via a variety of downstream transcription factors to finetune the regulation and complex process of OFT development.

## 5. Downstream Effectors of RA Signaling in Vertebrate Heart Development

The stimulation of RA signaling leads to the expression of downstream effectors that direct proper heart development. During early embryonic patterning, RA directly targets numerous homeobox (Hox) transcription factors that promote positional identity within embryos [141,142,143,144,145,146,147]. Data supports that anterior Hox genes are critical for proper OFT development in mice [148], which is consistent with the sensitivity of OFT defects observed from perturbed RA signaling. Lineage tracing experiments in mice have revealed that SHF progenitors contributing to both atria and the inferior wall of the OFT express *Hoxa1*, *Hoxa3*, and *Hoxb1*, all of which are RA-responsive [148]. Furthermore, enhancers for *Hoxb1* contain RAREs, implicating RA’s direct role in promoting Hoxb1 expression in the SHF and proepicardial progenitors [149], while *Hoxb1* KO mice have ventricular septal defects, abnormal positioning of the great arteries, and shorter OFTs due to increased proliferation and precocious differentiation of SHF progenitors [147]. Although *Hoxa1* KO mice do not have overt cardiac defects, loss of a single *Hoxb1* allele in *Hoxa1* mutants produces OFT defects similar to *Hoxb1* deficiency alone, supporting the notion there is some functional redundancy or compensation between these factors. In zebrafish, we have found that depletion of Hoxb5b, which is the RA-responsive within the ALPM, can specifically limit atrial specification [41]. However, these defects require confirmation in engineered mutants. We have found that excess Hox gene expression in zebrafish, due to the overexpression of the direct RA target Hoxb5b, strongly posteriorizes the ALPM in a manner analogous to excess RA treatment, and that high levels of Hoxb5b are capable of eliminating virtually all differentiated CMs [33,41]. Thus, Hox genes are likely key mediators of RA signaling that promote proper heart development during early patterning of the ALPM and slightly later during the accretion of SHF progenitors to the elongating heart tube.

Although data support that Hox genes are some of the key effectors of RA that mold the developing vertebrate heart, it is likely that the proper regulation of numerous transcription factors within a RA signaling gene regulatory network contributes to normal heart development. For instance, the orphan nuclear receptor subfamily 2 group F proteins (Nr2f; also called *Chicken ovalbumin upstream promoting transcription factors* (*Coup-TF*)) appears to function downstream of RA signaling within the ALPM. Nr2f1 and Nr2f2 have been shown to have conserved RA-responsiveness across vertebrate phyla [150,151,152,153,154,155,156]. Indeed, the requirement for Nr2f2 (Coup-TFII) in establishing atrial identity has been known for two decades, as Nr2f2 KO mice have atrial differentiation defects [157,158]. During the differentiation of atrial-like cardiomyocytes from human embryonic stem cells, NR2F1 and NR2F2 are upregulated upon treatment with exogenous RA [60,64]. Notably, while RA is part of protocols used to differentiate atrial-like cells from embryonic and human induced-pluripotent stem cells [60,61,62,64], as discussed above, a strict requirement for RA during atrial development in animal models has yet to support this role. Our recent study of Nr2f1a and Nr2f2 redundancy using engineered zebrafish mutants indicates that these Nr2fs are both required within the ALPM to restrict CM specification and promote posterior pharyngeal muscle differentiation [155]. In addition to forelimb defects, RA signaling deficient animals lose the posterior pharyngeal muscles, whose progenitors reside adjacent to cardiac progenitors [159,160]. Furthermore, detailed lineage tracing in mice has suggested that there may be bipotent populations of cardiopharyngeal progenitors, which contribute to both the heart and the pharyngeal muscles in mammals [161,162]. Our data imply that a RA-Nr2f regulatory network may contribute to cell fate choices between these differentiation paths downstream of RA signaling. 

## 6. Later Requirements of RA Signaling in Heart Development

**Figure 4:**
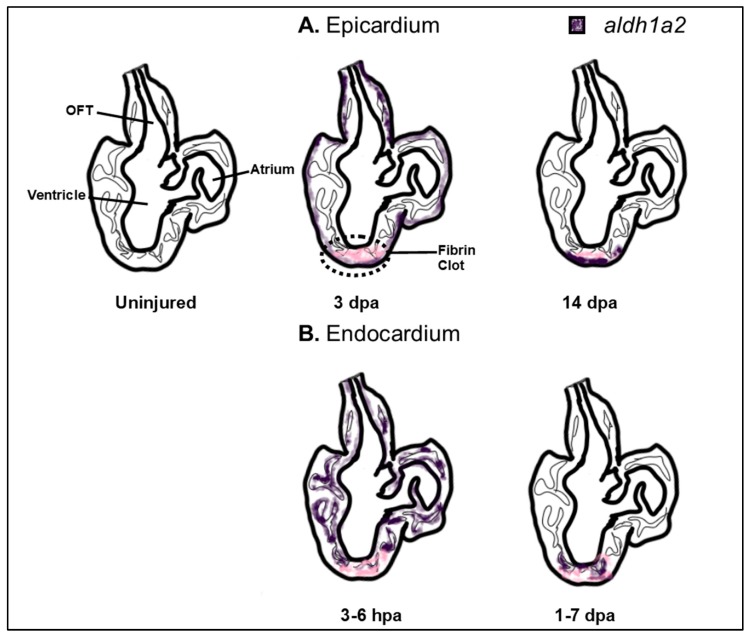
Cardiac proliferation and regenerative pathways are under the control of RA signaling. Schematic of *aldh1a2* expression in an apical resection model of adult zebrafish; however, a similar response is seen from other injuries and albeit subdued, in murine models [177]. In response to injury, enhanced *aldh1a2* expression is observed throughout the epicardium (**A**) and in the endocardium of both cardiac chambers (**B**). In the epicardium, *aldh1a2* expression becomes restricted to the site of injury, concomitant with deposition of the first layers of new muscle which require the coordination of RA-driven myocardial and epicardial events. Proper regeneration of the adult zebrafish heart requires RA synthesis from the endocardium as well, where RA synthesis rapidly becomes restricted to the site of injury as early as 24 h post-amputation. Dpa: day post-amputation, OFT: outflow tract.

Although many of the major structural defects from inappropriate RA signaling may be attributed to early defects in cardiac progenitor patterning and improper SHF development, RA also has roles in regulating later aspects of heart development (Figure 1). For instance, RA signaling is necessary for formation of the epicardium, diversification of epicardial-derived fibroblasts, and myocardial expansion [54,118,163,164,165]. Consistent with these requirements, *Aldh1a2* is observed by E11.5 and stage 18 in the epicardium of mouse and quail embryos, respectively [54,166]. In chicken embryos, maintenance of both *Wilm’s Tumor Gene 1* (*Wt1*) and *Aldh1a2* expression is necessary for the proper invasion of epicardial cells into the ventricular myocardium, while concomitantly limiting these cells from contributing to the coronary vasculature [167]. While Wt1 regulates an epithelial-to-mesenchymal transition (EMT) of the invading epicardial cells and promotes *Aldh1a2* expression in this population [164,168], its expression is also responsive to RA within epicardial cells, suggesting a feedback mechanism between both signals within this tissue. Both *Wt1* and RA signaling promote expression of the transcription factor Tcf21, which limits epicardial progenitors from differentiating into smooth muscle [165]. Notably, loss of *Tcf21* in mice leads to neonatal lethality with increased smooth muscle differentiation on the heart’s surface, epicardial blistering, and a paucity of interstitial fibroblasts [165]. During myocardial expansion in mammals, the epicardium produces a feedforward loop that is largely driven by the release of RA, which in turn induces hepatic erythropoietin to activate Igf2 [169,170]. Subsequently, CM proliferation proceeds through the upregulation of various growth pathways, including Pi3K/ERK, Fgf, and Wnt signaling [171,172,173,174]. Thus, many major pathways that control proliferation may be under the control of RA signaling. Interestingly in adult zebrafish, which can regenerate a significant portion of their heart, increased *aldh1a2* expression throughout the epicardium is among the first responses to injury. Inhibition of RA signaling in the epicardium and endocardium is sufficient to blunt proper regeneration of the adult zebrafish heart following resection of the ventricular apex [175,176,177] (Figure 4). The interplay of all of these pathways as downstream effectors of RA suggests that an improved understanding of RA signaling may have the potential to yield crucial therapeutic applications for cardiac regeneration.

## 7. Toward the Future: Understanding RA Signaling in the Vertebrate Heart

Proper levels of RA signaling are necessary for normal heart development in all vertebrates. Since the initial pivotal insights of Josef Warkany and his colleagues almost 70 years ago, significant progress has been made in our understanding of how proper RA signaling levels regulate distinct stages of vertebrate heart development. However, there is still a considerable way to go before many of these findings have translational therapeutic application. A particular deficiency in our current understanding is with respect to the larger gene regulatory networks that RA signaling employs to regulate heart development in each of the discussed contexts. Significant recent advances in sequencing techniques have led to a previously inconceivable ability to interrogate gene regulatory networks at the single cell level. Our enhanced ability to tease apart the specific RA-dependent epigenetic and transcriptional mechanisms that drive the gene regulatory networks at each stage of heart development will illuminate the intricacies of normal cardiac morphogenesis and the etiologies of congenital heart malformations in humans. Ultimately, we are hopeful that a new understanding is on the horizon that will lead to novel preventative and therapeutic strategies to confront the high incidence of CHDs. 

## Figures and Tables

**Table 1:** Cardiac defects arising from loss of RA signaling.

Cardiac Defect	Genes	Species	References
Increased CM specification/Cardiomegaly	*ALDH1A2*	Mouse	[28]
		Zebrafish	[65]
	*CYP26A1;CYP26C1*	Zebrafish	[42,44]
	*RARαb1*	Zebrafish	[34]
Outflow-tract defects	*CYP26A1*	Human	[72,73]
	*DHRS3*	Mouse	[26,74]
	*STRA6*	Human	[75,76,77]
	*RARα1;RARβ*		
	*RARα1;RARγ*		
	*RARα1;RXRα*	Mouse	[35,78,79]
	*RARβ1;RXRα*		
	*RARβ2;RXRα*		
Atrioventricular septal defects	*DHRS3*	Mouse	[74]
	*STRA6*	Human	[76]
Asymmetry/ Looping defects	*ALDH1A2*	Mouse	[28,53,54,55,67,68]
	*CYP26A1*	Mouse	[80,81,82]
	*CYP26A1;CYP26C1*	Mouse	[83]
	*RARγ*	Chicken	[84]
	*RDH10*	Mouse	[85,86,87]
	*STRA6*	Human	[88]
Inflow-tract defects	*RARα2*	Chicken	[84]

## References

[1] Van der Linde D., Konings E.E.M., Slager M.A., Witsenburg M., Helbing W.A., Takkenberg J.J.M., Roos-Hesselink J.W. (2011). Birth Prevalence of Congenital Heart Disease Worldwide: A Systematic Review and Meta-Analysis. J. Am. Coll. Cardiol..

[2] Benjamin E.J., Muntner P., Alonso A., Bittencourt M.S., Callaway C.W., Carson A.P., Chamberlain A.M., Chang A.R., Cheng S., Das S.R. (2019). Heart Disease and Stroke Statistics-2019 Update: A Report From the American Heart Association. Circulation.

[3] D’Aniello E., Waxman J.S. (2015). Input overload: Contributions of retinoic acid signaling feedback mechanisms to heart development and teratogenesis. Dev. Dyn..

[4] Stefanovic S., Zaffran S. (2017). Mechanisms of retinoic acid signaling during cardiogenesis. Mech. Dev..

[5] Wilson J.G., Roth C.B., Warkany J. (1953). An analysis of the syndrome of malformations induced by maternal vitamin a deficiency. Effects of restoration of vitamin a at various times during gestation. Am. J. Anat..

[6] Wilson J.G., Warkany J. (1950). Cardiac and aortic arch anomalies in the offspring of vitamin A deficient rats correlated with similar human anomalies. Pediatrics.

[7] Dersch H., Zile M.H. (1993). Induction of Normal Cardiovascular Development in the Vitamin A-Deprived Quail Embryo by Natural Retinoids. Dev. Biol..

[8] Zile M.H. (2010). Vitamin A-not for your eyes only: Requirement for heart formation begins early in embryogenesis. Nutrients.

[9] Finnell R.H., Shaw G.M., Lammer E.J., Brandl K.L., Carmichael S.L., Rosenquist T.H. (2004). Gene–nutrient interactions: importance of folates and retinoids during early embryogenesis. Toxicol. Appl. Pharmacol..

[10] World Health Organization (2009). Global Prevalence of Vitamin A Deficiency in Populations at Risk 1995–2005: WHO Global Database on Vitamin A Deficiency.

[11] Lammer E.J., Chen D.T., Hoar R.M., Agnish N.D., Benke P.J., Braun J.T., Curry C.J., Fernhoff P.M., Grix A.W., Lott I.T. (1985). Retinoic Acid Embryopathy. N. Engl. J. Med..

[12] Mondal D., R Shenoy S., Mishra S. (2017). Retinoic acid embryopathy. Int. J. Appl. Basic Med. Res..

[13] Doshi A. (2007). The cost of clear skin: balancing the social and safety costs of iPLEDGE with the efficacy of Accutane (isotretinoin). Seton Hall Law Rev..

[14] Duerbeck N.B., Dowling D.D. (2012). Vitamin A: Too much of a good thing?. Obstet. Gynecol. Surv..

[15] Wolbach S.B., Howe P.R. (1925). Tissue changes following deprivation of fat-soluble A vitamin. J. Exp. Med..

[16] Hale F. (1935). The Relation of Vitamin a to Anophthalmos in Pigs. Am. J. Ophthalmol..

[17] Wilson J.G., Warkany J. (1949). Aortic-arch and cardiac anomalies in the offspring of vitamin A deficient rats. Am. J. Anat..

[18] Wilson J.G., Warkany J. (1950). Congenital anomalies of heart and great vessels in offspring of vitamin A-deficient rats. Am. J. Dis. Child..

[19] Cohlan S.Q. (1953). Excessive intake of vitamin A as a cause of congenital anomalies in the rat. Science.

[20] Kalter H., Warkany J. (1961). Experimental production of congenital malformations in strains of inbred mice by maternal treatment with hypervitaminosis A. Am. J. Pathol..

[21] Pan J., Baker K.M. (2007). Retinoic Acid and the Heart. Vitam. Horm..

[22] Rizzo R., Lammer E.J., Parano E., Pavone L., Argyle J.C. (1991). Limb reduction defects in humans associated with prenatal isotretinoin exposure. Teratology.

[23] Rothman K.J., Moore L.L., Singer M.R., Nguyen U.-S.D.T., Mannino S., Milunsky A. (1995). Teratogenicity of High Vitamin A Intake. N. Engl. J. Med..

[24] Gliniak C.M., Brown J.M., Noy N. (2017). The retinol-binding protein receptor STRA6 regulates diurnal insulin responses. J. Biol. Chem..

[25] Heine U.I., Roberts A.B., Munoz E.F., Roche N.S., Sporn M.B. (1985). Effects of retinoid deficiency on the development of the heart and vascular system of the quail embryo. Virchows Arch. B. Cell Pathol. Incl. Mol. Pathol..

[26] Wang S., Huang W., Castillo H.A., Kane M.A., Xavier-Neto J., Trainor P.A., Moise A.R. (2018). Alterations in retinoic acid signaling affect the development of the mouse coronary vasculature. Dev. Dyn..

[27] Sugrue K.F., Sarkar A.A., Leatherbury L., Zohn I.E. (2018). The ubiquitin ligase HECTD1 promotes retinoic acid signaling required for development of the aortic arch. Dis. Model. Mech..

[28] Niederreither K., Subbarayan V., Dollé P., Chambon P. (1999). Embryonic retinoic acid synthesis is essential for early mouse post-implantation development. Nat. Genet..

[29] Stainier D.Y.R., Fishman M.C. (1992). Patterning the zebrafish heart tube: Acquisition of anteroposterior polarity. Dev. Biol..

[30] Yutzey K.E., Rhee J.T., Bader D. (1994). Expression of the atrial-specific myosin heavy chain {AMHC1} and the establishment of anteroposterior polarity in the developing chicken heart. Development.

[31] Kostetskii I., Yuan S.-Y., Kostetskaia E., Linask K.K., Blanchet S., Seleiro E., Michaille J.-J., Brickell P., Zile M. (1998). Initial retinoid requirement for early avian development coincides with retinoid receptor coexpression in the precardiac fields and induction of normal cardiovascular development. Dev. Dyn..

[32] LaRue A.C., Argraves W.S., Zile M.H., Drake C.J. (2004). Critical role for retinol in the generation/differentiation of angioblasts required for embryonic blood vessel formation. Dev. Dyn..

[33] Waxman J.S., Yelon D. (2009). Increased Hox activity mimics the teratogenic effects of excess retinoic acid signaling. Dev. Dyn..

[34] D’Aniello E., Rydeen A.B., Anderson J.L., Mandal A., Waxman J.S. (2013). Depletion of Retinoic Acid Receptors Initiates a Novel Positive Feedback Mechanism that Promotes Teratogenic Increases in Retinoic Acid. PLoS Genet..

[35] Mendelsohn C., Lohnes D., Décimo D., Lufkin T., LeMeur M., Chambon P., Mark M. (1994). Function of the retinoic acid receptors (RARs) during development (II). Multiple abnormalities at various stages of organogenesis in RAR double mutants. Development.

[36] Dickman E.D., Thaller C., Smith S.M. (1997). Temporally-regulated retinoic acid depletion produces specific neural crest, ocular and nervous system defects. Development.

[37] Duester G. (2008). Retinoic Acid Synthesis and Signaling during Early Organogenesis. Cell.

[38] Niederreither K., Dollé P. (2008). Retinoic acid in development: Towards an integrated view. Nat. Rev. Genet..

[39] Liu J., Stainier D.Y.R. (2012). Zebrafish in the Study of Early Cardiac Development. Circ. Res..

[40] Schilling T.F., Nie Q., Lander A.D. (2012). Dynamics and precision in retinoic acid morphogen gradients. Curr. Opin. Genet. Dev..

[41] Waxman J.S., Keegan B.R., Roberts R.W., Poss K.D., Yelon D. (2008). Hoxb5b Acts Downstream of Retinoic Acid Signaling in the Forelimb Field to Restrict Heart Field Potential in Zebrafish. Dev. Cell.

[42] Rydeen A.B., Waxman J.S. (2016). Cyp26 Enzymes Facilitate Second Heart Field Progenitor Addition and Maintenance of Ventricular Integrity. PLoS Biol..

[43] De Bono C., Thellier C., Bertrand N., Sturny R., Jullian E., Cortes C., Stefanovic S., Zaffran S., Théveniau-Ruissy M., Kelly R.G. (2018). T-box genes and retinoic acid signaling regulate the segregation of arterial and venous pole progenitor cells in the murine second heart field. Hum. Mol. Genet..

[44] Rydeen A.B., Waxman J.S. (2014). Cyp26 enzymes are required to balance the cardiac and vascular lineages within the anterior lateral plate mesoderm. Development.

[45] Liu Q., Van Bortle K., Zhang Y., Zhao M., Zhang J.Z., Geller B.S., Gruber J.J., Jiang C., Wu J.C., Snyder M.P. (2018). Disruption of mesoderm formation during cardiac differentiation due to developmental exposure to 13-cis-retinoic acid. Sci. Rep..

[46] Bakkers J. (2011). Zebrafish as a model to study cardiac development and human cardiac disease. Cardiovasc. Res..

[47] Staudt D., Stainier D. (2012). Uncovering the Molecular and Cellular Mechanisms of Heart Development Using the Zebrafish. Annu. Rev. Genet..

[48] Santini M.P., Forte E., Harvey R.P., Kovacic J.C. (2016). Developmental origin and lineage plasticity of endogenous cardiac stem cells. Development.

[49] Laugwitz K.-L., Moretti A., Caron L., Nakano A., Chien K.R. (2007). Islet1 cardiovascular progenitors: A single source for heart lineages?. Development.

[50] Buckingham M. (2016). First and Second Heart Field. Congenital Heart Diseases: The Broken Heart.

[51] Durston A.J., Timmermans J.P.M., Hage W.J., Hendriks H.F.J., de Vries N.J., Heideveld M., Nieuwkoop P.D. (1989). Retinoic acid causes an anteroposterior transformation in the developing central nervous system. Nature.

[52] Sive H.L., Draper B.W., Harland R.M., Weintraub H. (1990). Identification of a retinoic acid-sensitive period during primary axis formation in Xenopus laevis. Genes Dev..

[53] Hochgreb T. (2003). A caudorostral wave of RALDH2 conveys anteroposterior information to the cardiac field. Development.

[54] Moss J.B., Xavier-Neto J., Shapiro M.D., Nayeem S.M., McCaffery P., Dräger U.C., Rosenthal N. (1998). Dynamic Patterns of Retinoic Acid Synthesis and Response in the Developing Mammalian Heart. Dev. Biol..

[55] Niederreither K., Fraulob V., Garnier J.-M., Chambon P., Dollé P. (2002). Differential expression of retinoic acid-synthesizing (RALDH) enzymes during fetal development and organ differentiation in the mouse. Mech. Dev..

[56] Xavier-Neto J., Rosenthal N., Silva F.A., Matos T.G.F., Hochgreb T., Linhares V.L.F. (2001). Retinoid signaling and cardiac anteroposterior segmentation. Genesis.

[57] Simões-Costa M.S., Vasconcelos M., Sampaio A.C., Cravo R.M., Linhares V.L., Hochgreb T., Yan C.Y.I., Davidson B., Xavier-Neto J. (2005). The evolutionary origin of cardiac chambers. Dev. Biol..

[58] Osmond M.K., Butler A.J., Voon F.C.T., Bellairs R. (1991). The effects of retinoic acid on heart formation in the early chick embryo. Development.

[59] Xavier-Neto J., Neville C., Shapiro M., Houghton L., Wang G., Nikovits W., Stockdale F., Rosenthal N. (1999). A retinoic acid-inducible transgenic marker of sino-atrial development in the mouse heart. Development.

[60] Devalla H.D., Schwach V., Ford J.W., Milnes J.T., El-Haou S., Jackson C., Gkatzis K., Elliott D.A., Chuva de Sousa Lopes S.M., Mummery C.L. (2015). Atrial-like cardiomyocytes from human pluripotent stem cells are a robust preclinical model for assessing atrial-selective pharmacology. EMBO Mol. Med..

[61] Zhang Q., Jiang J., Han P., Yuan Q., Zhang J., Zhang X., Xu Y., Cao H., Meng Q., Chen L. (2011). Direct differentiation of atrial and ventricular myocytes from human embryonic stem cells by alternating retinoid signals. Cell Res..

[62] Gassanov N., Er F., Zagidullin N., Jankowski M., Gutkowska J., Hoppe U.C. (2008). Retinoid acid-induced effects on atrial and pacemaker cell differentiation and expression of cardiac ion channels. Differentiation.

[63] Lee J.H., Protze S.I., Laksman Z., Backx P.H., Keller G.M. (2017). Human Pluripotent Stem Cell-Derived Atrial and Ventricular Cardiomyocytes Develop from Distinct Mesoderm Populations. Cell Stem Cell.

[64] Quaranta R., Fell J., Rühle F., Rao J., Piccini I., Araúzo-Bravo M.J., Verkerk A.O., Stoll M., Greber B. (2018). Revised roles of ISL1 in a hES cell-based model of human heart chamber specification. Elife.

[65] Keegan B.R., Feldman J.L., Begemann G., Ingham P.W., Yelon D. (2005). Retinoic acid signaling restricts the cardiac progenitor pool. Science.

[66] Collop A.H., Broomfield J.A.S., Chandraratna R.A.S., Yong Z., Deimling S.J., Kolker S.J., Weeks D.L., Drysdale T.A. (2006). Retinoic acid signaling is essential for formation of the heart tube in Xenopus. Dev. Biol..

[67] Ryckebusch L., Wang Z., Bertrand N., Lin S.-C., Chi X., Schwartz R., Zaffran S., Niederreither K. (2008). Retinoic acid deficiency alters second heart field formation. Proc. Natl. Acad. Sci. USA.

[68] Sirbu I.O., Zhao X., Duester G. (2008). Retinoic acid controls heart anteroposterior patterning by down-regulatingIsl1 through theFgf8 pathway. Dev. Dyn..

[69] Keegan B.R., Meyer D., Yelon D. (2004). Organization of cardiac chamber progenitors in the zebrafish blastula. Development.

[70] Schoenebeck J.J., Keegan B.R., Yelon D. (2007). Vessel and Blood Specification Override Cardiac Potential in Anterior Mesoderm. Dev. Cell.

[71] Abu-Issa R., Kirby M.L. (2008). Patterning of the heart field in the chick. Dev. Biol..

[72] Roberts C., Ivins S., Cook A.C., Baldini A., Scambler P.J. (2006). Cyp26 genes a1, b1 and c1 are down-regulated in Tbx1 null mice and inhibition of Cyp26 enzyme function produces a phenocopy of DiGeorge Syndrome in the chick. Hum. Mol. Genet..

[73] Pennimpede T., Cameron D.A., MacLean G.A., Li H., Abu-Abed S., Petkovich M. (2010). The role of CYP26 enzymes in defining appropriate retinoic acid exposure during embryogenesis. Birth Defects Res. Part A Clin. Mol. Teratol..

[74] Billings S.E., Pierzchalski K., Butler Tjaden N.E., Pang X.-Y., Trainor P.A., Kane M.A., Moise A.R. (2013). The retinaldehyde reductase DHRS3 is essential for preventing the formation of excess retinoic acid during embryonic development. FASEB J..

[75] Pasutto F., Sticht H., Hammersen G., Gillessen-Kaesbach G., FitzPatrick D.R., Nürnberg G., Brasch F., Schirmer-Zimmermann H., Tolmie J.L., Chitayat D. (2007). Mutations in STRA6 Cause a Broad Spectrum of Malformations Including Anophthalmia, Congenital Heart Defects, Diaphragmatic Hernia, Alveolar Capillary Dysplasia, Lung Hypoplasia, and Mental Retardation. Am. J. Hum. Genet..

[76] Golzio C., Martinovic-Bouriel J., Thomas S., Mougou-Zrelli S., Grattagliano-Bessières B., Bonnière M., Delahaye S., Munnich A., Encha-Razavi F., Lyonnet S. (2007). Matthew-Wood Syndrome Is Caused by Truncating Mutations in the Retinol-Binding Protein Receptor Gene STRA6. Am. J. Hum. Genet..

[77] Noy N. (2016). Vitamin a transport and cell Signaling by the retinol-binding protein receptor STRA6. Sub-Cellular Biochemistry.

[78] Ghyselinck N., Wendling O., Messaddeq N., Dierich A., Lampron C., Décimo D., Viville S., Chambon P., Mark M. (1998). Contribution of retinoic acid receptor β isoforms to the formation of the conotruncal septum of the embryonic heart. Dev. Biol..

[79] Li P., Pashmforoush M., Sucov H.M. (2010). Retinoic acid regulates differentiation of the secondary heart field and TGFbeta-mediated outflow tract septation. Dev. Cell.

[80] Abu-Abed S., Dollé P., Metzger D., Beckett B., Chambon P., Petkovich M. (2001). The retinoic acid-metabolizing enzyme, CYP26A1, is essential for normal hindbrain patterning, vertebral identity, and development of posterior structures. Genes Dev..

[81] Kudoh T., Wilson S.W., Dawid I.B. (2002). Distinct roles for Fgf, Wnt and retinoic acid in posteriorizing the neural ectoderm. Development.

[82] Emoto Y., Wada H., Okamoto H., Kudo A., Imai Y. (2005). Retinoic acid-metabolizing enzyme Cyp26a1 is essential for determining territories of hindbrain and spinal cord in zebrafish. Dev. Biol..

[83] Uehara M., Yashiro K., Mamiya S., Nishino J., Chambon P., Dolle P., Sakai Y. (2007). CYP26A1 and CYP26C1 cooperatively regulate anterior–posterior patterning of the developing brain and the production of migratory cranial neural crest cells in the mouse. Dev. Biol..

[84] Romeih M., Cui J., Michaille J.-J., Jiang W., Zile M.H. (2003). Function of RARgamma and RARalpha2 at the initiation of retinoid signaling is essential for avian embryo survival and for distinct events in cardiac morphogenesis. Dev. Dyn..

[85] Sandell L.L., Sanderson B.W., Moiseyev G., Johnson T., Mushegian A., Young K., Rey J.-P., Ma J.-X., Staehling-Hampton K., Trainor P.A. (2007). RDH10 is essential for synthesis of embryonic retinoic acid and is required for limb, craniofacial, and organ development. Genes Dev..

[86] Sandell L.L., Lynn M.L., Inman K.E., McDowell W., Trainor P.A. (2012). RDH10 Oxidation of Vitamin A Is a Critical Control Step in Synthesis of Retinoic Acid during Mouse Embryogenesis. PLoS ONE.

[87] Rhinn M., Schuhbaur B., Niederreither K., Dolle P. (2011). Involvement of retinol dehydrogenase 10 in embryonic patterning and rescue of its loss of function by maternal retinaldehyde treatment. Proc. Natl. Acad. Sci. USA.

[88] Cubuk P.O., Ho L., Reversade B., Perçin E.F. (2016). MATTHEW-WOOD SYNDROME: A CASE WITH DEXTROCARDIA AND STREAK GONADS. Genet. Couns..

[89] Dollé P., Niederreither K., Dollé P., Neiderreither K. (2015). The Retinoids.

[90] Zhao X., Sirbu I.O., Mic F.A., Molotkova N., Molotkov A., Kumar S., Duester G. (2009). Retinoic Acid Promotes Limb Induction through Effects on Body Axis Extension but Is Unnecessary for Limb Patterning. Curr. Biol..

[91] Cunningham T.J., Zhao X., Sandell L.L., Evans S.M., Trainor P.A., Duester G. (2013). Antagonism between Retinoic Acid and Fibroblast Growth Factor Signaling during Limb Development. Cell Rep..

[92] Sorrell M.R.J., Waxman J.S. (2011). Restraint of Fgf8 signaling by retinoic acid signaling is required for proper heart and forelimb formation. Dev. Biol..

[93] Yi Li Q., Newbury-Ecob R.A., Terrett J.A., Wilson D.I., Curtis A.R.J., Ho Yi C., Gebuhr T., Bullen P.J., Robson S.C., Strachan T. (1997). Holt-Oram syndrome is caused by mutations in TBX5, a member of the Brachyury (T) gene family. Nat. Genet..

[94] Basson C.T., Bachinsky D.R., Lin R.C., Levi T., Elkins J.A., Soults J., Grayzel D., Kroumpouzou E., Traill T.A., Leblanc-Straceski J. (1997). Mutations in human cause limb and cardiac malformation in Holt-Oram syndrome. Nat. Genet..

[95] Duong T.B., Ravisankar P., Song Y.C., Gafranek J.T., Rydeen A.B., Dohn T.E., Barske L.A., Crump J.G., Waxman J.S. (2018). Nr2f1a balances atrial chamber and atrioventricular canal size via BMP signaling-independent and -dependent mechanisms. Dev. Biol..

[96] Barski A., Zhao K. (2009). Genomic location analysis by ChIP-Seq. J. Cell. Biochem..

[97] Kumar S., Duester G. (2014). Retinoic acid controls body axis extension by directly repressing Fgf8 transcription. Development.

[98] Kumar S., Cunningham T.J., Duester G. (2016). Nuclear receptor corepressors Ncor1 and Ncor2 (Smrt) are required for retinoic acid-dependent repression of Fgf8 during somitogenesis. Dev. Biol..

[99] Rudnicki M.A., McBurney M.W., Robertson E. (1987). Teratocarcinomas and Embryonic Stem Cells: A practical Approach.

[100] Edwards M.K., McBurney M.W. (1983). The concentration of retinoic acid determines the differentiated cell types formed by a teratocarcinoma cell line. Dev. Biol..

[101] Wobus A.M., Rohwedel J., Maltsev V., Hescheler J. (1994). In vitro differentiation of embryonic stem cells into cardiomyocytes or skeletal muscle cells is specifically modulated by retinoic acid. Roux’s Arch. Dev. Biol..

[102] Hernandez R.E., Putzke A.P., Myers J.P., Margaretha L., Moens C.B. (2007). Cyp26 enzymes generate the retinoic acid response pattern necessary for hindbrain development. Development.

[103] Samarut E., Fraher D., Gibert Y. (2015). ZebRA: An overview of retinoic acid signaling during zebrafish development. Biochim. Biophys. Acta Gene Regul. Mech..

[104] White R.J., Nie Q., Lander A.D., Schilling T.F. (2007). Complex Regulation of cyp26a1 Creates a Robust Retinoic Acid Gradient in the Zebrafish Embryo. PLoS Biol..

[105] Sosnik J., Zheng L., Rackauckas C.V., Digman M., Gratton E., Nie Q., Schilling T.F. (2016). Noise modulation in retinoic acid signaling sharpens segmental boundaries of gene expression in the embryonic zebrafish hindbrain. Elife.

[106] Shimozono S., Iimura T., Kitaguchi T., Higashijima S.-I., Miyawaki A. (2013). Visualization of an endogenous retinoic acid gradient across embryonic development. Nature.

[107] Ruiz A., Mark M., Jacobs H., Klopfenstein M., Hu J., Lloyd M., Habib S., Tosha C., Radu R.A., Ghyselinck N.B. (2012). Retinoid content, visual responses, and ocular morphology are compromised in the retinas of mice lacking the retinol-binding protein receptor, STRA6. Investig. Ophthalmol. Vis. Sci..

[108] Berry D.C., Jacobs H., Marwarha G., Gely-Pernot A., O’Byrne S.M., DeSantis D., Klopfenstein M., Feret B., Dennefeld C., Blaner W.S. (2013). The STRA6 receptor is essential for retinol-binding protein-induced insulin resistance but not for maintaining vitamin A homeostasis in tissues other than the eye. J. Biol. Chem..

[109] Amengual J., Zhang N., Kemerer M., Maeda T., Palczewski K., Von Lintig J. (2014). STRA6 is critical for cellular vitamin A uptake and homeostasis. Hum. Mol. Genet..

[110] Quadro L., Blaner W.S., Salchow D.J., Vogel S., Piantedosi R., Gouras P., Freeman S., Cosma M.P., Colantuoni V., Gottesman M.E. (1999). Impaired retinal function and vitamin A availability in mice lacking retinol-binding protein. EMBO J..

[111] Batten M.L., Imanishi Y., Maeda T., Tu D.C., Moise A.R., Bronson D., Possin D., Van Gelder R.N., Baehr W., Palczewski K. (2004). Lecithin-retinol Acyltransferase Is Essential for Accumulation of All- *trans* -Retinyl Esters in the Eye and in the Liver. J. Biol. Chem..

[112] Kedishvili N.Y. (2013). Enzymology of retinoic acid biosynthesis and degradation. J. Lipid Res..

[113] Chatzi C., Cunningham T.J., Duester G. (2013). Investigation of retinoic acid function during embryonic brain development using retinaldehyde-rescued Rdh10 knockout mice. Dev. Dyn..

[114] D’Aniello E., Ravisankar P., Waxman J.S. (2015). Rdh10a Provides a Conserved Critical Step in the Synthesis of Retinoic Acid during Zebrafish Embryogenesis. PLoS ONE.

[115] Ryckebüsch L., Bertrand N., Mesbah K., Bajolle F., Niederreither K., Kelly R.G., Zaffran S. (2010). Decreased levels of embryonic retinoic acid synthesis accelerate recovery from arterial growth delay in a mouse model of DiGeorge syndrome. Circ. Res..

[116] Théveniau-Ruissy M., Dandonneau M., Mesbah K., Ghez O., Mattei M.-G., Miquerol L., Kelly R.G. (2008). The del22q11.2 Candidate Gene *Tbx1* Controls Regional Outflow Tract Identity and Coronary Artery Patterning. Circ. Res..

[117] Rana M.S., Théveniau-Ruissy M., De Bono C., Mesbah K., Francou A., Rammah M., Domínguez J.N., Roux M., Laforest B., Anderson R.H. (2014). Tbx1 Coordinates Addition of Posterior Second Heart Field Progenitor Cells to the Arterial and Venous Poles of the Heart. Circ. Res..

[118] El Robrini N., Etchevers H.C., Ryckebüsch L., Faure E., Eudes N., Niederreither K., Zaffran S., Bertrand N. (2016). Cardiac outflow morphogenesis depends on effects of retinoic acid signaling on multiple cell lineages. Dev. Dyn..

[119] Dobbs-McAuliffe B., Zhao Q., Linney E. (2004). Feedback mechanisms regulate retinoic acid production and degradation in the zebrafish embryo. Mech. Dev..

[120] Yagi H., Furutani Y., Hamada H., Sasaki T., Asakawa S., Minoshima S., Ichida F., Joo K., Kimura M., Imamura S. (2003). Role of TBX1 in human del22q11.2 syndrome. Lancet.

[121] Guris D.L., Duester G., Papaioannou V.E., Imamoto A. (2006). Dose-Dependent Interaction of Tbx1 and Crkl and Locally Aberrant RA Signaling in a Model of del22q11 Syndrome. Dev. Cell.

[122] Roberts C., Ivins S.M., James C.T., Scambler P.J. (2005). Retinoic acid down-regulatesTbx1 expression in vivo and in vitro. Dev. Dyn..

[123] Maynard T.M., Gopalakrishna D., Meechan D.W., Paronett E.M., Newbern J.M., LaMantia A.-S. (2013). 22q11 Gene dosage establishes an adaptive range for sonic hedgehog and retinoic acid signaling during early development. Hum. Mol. Genet..

[124] Niederreither K., Abu-Abed S., Schuhbaur B., Petkovich M., Chambon P., Dollé P. (2002). Genetic evidence that oxidative derivatives of retinoic acid are not involved in retinoid signaling during mouse development. Nat. Genet..

[125] Sakai Y., Meno C., Fujii H., Nishino J., Shiratori H., Saijoh Y., Rossant J., Hamada H. (2001). The retinoic acid-inactivating enzyme CYP26 is essential for establishing an uneven distribution of retinoic acid along the anterio-posterior axis within the mouse embryo. Genes Dev..

[126] Adams M.K., Belyaeva O.V., Wu L., Kedishvili N.Y. (2014). The retinaldehyde reductase activity of DHRS3 is reciprocally activated by retinol dehydrogenase 10 to control retinoid homeostasis. J. Biol. Chem..

[127] Feng L., Hernandez R.E., Waxman J.S., Yelon D., Moens C.B. (2010). Dhrs3a regulates retinoic acid biosynthesis through a feedback inhibition mechanism. Dev. Biol..

[128] Bastien J., Rochette-Egly C. (2004). Nuclear retinoid receptors and the transcription of retinoid-target genes. Gene.

[129] Dupé V., Ghyselinck N.B., Wendling O., Chambon P., Mark M. (1999). Key roles of retinoic acid receptors alpha and beta in the patterning of the caudal hindbrain, pharyngeal arches and otocyst in the mouse. Development.

[130] Sucov H.M., Dyson E., Gumeringer C.L., Price J., Chien K.R., Evans R.M. (1994). RXR alpha mutant mice establish a genetic basis for vitamin A signaling in heart morphogenesis. Genes Dev..

[131] Gruber P.J., Kubalak S.W., Pexieder T., Sucov H.M., Evans R.M., Chien K.R. (1996). RXR alpha deficiency confers genetic susceptibility for aortic sac, conotruncal, atrioventricular cushion, and ventricular muscle defects in mice. J. Clin. Investig..

[132] Kastner P., Grondona J.M., Mark M., Gansmuller A., LeMeur M., Decimo D., Vonesch J.-L., Dollé P., Chambon P. (1994). Genetic analysis of RXRα developmental function: Convergence of RXR and RAR signaling pathways in heart and eye morphogenesis. Cell.

[133] Li E., Sucov H.M., Lee K.F., Evans R.M., Jaenisch R. (1993). Normal development and growth of mice carrying a targeted disruption of the alpha 1 retinoic acid receptor gene. Proc. Natl. Acad. Sci. USA.

[134] Kastner P., Messaddeq N., Mark M., Wendling O., Grondona J.M., Ward S., Ghyselinck N., Chambon P. (1997). Vitamin A deficiency and mutations of RXRalpha, RXRbeta and RARalpha lead to early differentiation of embryonic ventricular cardiomyocytes. Development.

[135] Chen J., Kubalak S.W., Chien K.R. (1998). Ventricular muscle-restricted targeting of the RXRalpha gene reveals a non-cell-autonomous requirement in cardiac chamber morphogenesis. Development.

[136] Xin M., Davis C.A., Molkentin J.D., Lien C.-L., Duncan S.A., Richardson J.A., Olson E.N. (2006). A threshold of GATA4 and GATA6 expression is required for cardiovascular development. Proc. Natl. Acad. Sci. USA.

[137] Sakabe M., Kokubo H., Nakajima Y., Saga Y. (2012). Ectopic retinoic acid signaling affects outflow tract cushion development through suppression of the myocardial Tbx2-Tgf 2 pathway. Development.

[138] Bertrand S., Thisse B., Tavares R., Sachs L., Chaumot A., Bardet P.-L., Escrivà H., Duffraisse M., Marchand O., Safi R. (2007). Unexpected Novel Relational Links Uncovered by Extensive Developmental Profiling of Nuclear Receptor Expression. PLoS Genet..

[139] Waxman J.S., Yelon D. (2007). Comparison of the expression patterns of newly identified zebrafish retinoic acid and retinoid X receptors. Dev. Dyn..

[140] Linville A., Radtke K., Waxman J.S., Yelon D., Schilling T.F. (2009). Combinatorial roles for zebrafish retinoic acid receptors in the hindbrain, limbs and pharyngeal arches. Dev. Biol..

[141] Huang D., Chen S., Langston A., Gudas L. (1998). A conserved retinoic acid responsive element in the murine Hoxb-1 gene is required for expression in the developing gut. Development.

[142] Marshall H., Studer M., Pöpperl H., Aparicio S., Kuroiwa A., Brenner S., Krumlauf R. (1994). A conserved retinoic acid response element required for early expression of the homeobox gene Hoxb-1. Nature.

[143] Oosterveen T., van Vliet P., Deschamps J., Meijlink F. (2003). The Direct Context of a Hox Retinoic Acid Response Element Is Crucial for its Activity. J. Biol. Chem..

[144] Langston A.W., Thompson J.R., Gudas L.J. (1997). Retinoic Acid-responsive Enhancers Located 3′ of the Hox A and Hox B Homeobox Gene Clusters. J. Biol. Chem..

[145] LaRosa G.J., Gudas L.J. (1988). Early retinoic acid-induced F9 teratocarcinoma stem cell gene ERA-1: alternate splicing creates transcripts for a homeobox-containing protein and one lacking the homeobox. Mol. Cell. Biol..

[146] Dupé V., Davenne M., Brocard J., Dollé P., Mark M., Dierich A., Chambon P., Rijli F.M. (1997). In vivo functional analysis of the Hoxa-1 3’ retinoic acid response element (3’RARE). Development.

[147] Roux M., Laforest B., Capecchi M., Bertrand N., Zaffran S. (2015). Hoxb1 regulates proliferation and differentiation of second heart field progenitors in pharyngeal mesoderm and genetically interacts with Hoxa1 during cardiac outflow tract development. Dev. Biol..

[148] Bertrand N., Roux M., Ryckebüsch L., Niederreither K., Dollé P., Moon A., Capecchi M., Zaffran S. (2011). Hox genes define distinct progenitor sub-domains within the second heart field. Dev. Biol..

[149] Nolte C., Jinks T., Wang X., Martinez Pastor M.T., Krumlauf R. (2013). Shadow enhancers flanking the HoxB cluster direct dynamic Hox expression in early heart and endoderm development. Dev. Biol..

[150] Jonk L.J.C., de Jonge M.E.J., Vervaart J.M.A., Wissink S., Kruijer W. (1994). Isolation and Developmental Expression of Retinoic-Acid-Induced Genes. Dev. Biol..

[151] van der Wees J., Matharu P.J., de Roos K., Destre´e O.H.J., Godsave S.F., Durston A.J., Sweeney G.E. (1996). Developmental expression and differential regulation by retinoic acid ofXenopus COUP-TF-A andCOUP-TF-B. Mech. Dev..

[152] Pereira F.A., Tsai M.J., Tsai S.Y. (2000). COUP-TF orphan nuclear receptors in development and differentiation. Cell. Mol. Life Sci..

[153] Love C.E., Prince V.E. (2012). Expression and retinoic acid regulation of the zebrafish *nr2f* orphan nuclear receptor genes. Dev. Dyn..

[154] Laursen K.B., Mongan N.P., Zhuang Y., Ng M.M., Benoit Y.D., Gudas L.J. (2013). Polycomb recruitment attenuates retinoic acid–induced transcription of the bivalent NR2F1 gene. Nucleic Acids Res..

[155] Dohn T.E., Ravisankar P., Tirera F.T., Martin K.E., Gafranek J.T., Duong T.B., VanDyke T.L., Touvron M., Barske L.A., Crump J.G. (2019). Nr2f-dependent allocation of ventricular cardiomyocyte and pharyngeal muscle progenitors. PLoS Genet..

[156] Lin F.-J., Qin J., Tang K., Tsai S.Y., Tsai M.-J. (2011). Coup d’Etat: An Orphan Takes Control. Endocr. Rev..

[157] Pereira F.A., Qiu Y., Zhou G., Tsai M.-J., Tsai S.Y. (1999). The orphan nuclear receptor COUP-TFII is required for angiogenesis and heart development. Genes Dev..

[158] Wu S.P., Cheng C.M., Lanz R.B., Wang T., Respress J.L., Ather S., Chen W., Tsai S.J., Wehrens X.H.T., Tsai M.J. (2013). Atrial Identity Is Determined by a COUP-TFII Regulatory Network. Dev. Cell.

[159] Begemann G., Schilling T.F., Rauch G.J., Geisler R., Ingham P.W. (2001). The zebrafish neckless mutation reveals a requirement for raldh2 in mesodermal signals that pattern the hindbrain. Development.

[160] Niederreither K. (2003). The regional pattern of retinoic acid synthesis by RALDH2 is essential for the development of posterior pharyngeal arches and the enteric nervous system. Development.

[161] Meilhac S.M., Lescroart F., Blanpain C., Buckingham M.E. (2014). Cardiac cell lineages that form the heart. Cold Spring Harb. Perspect. Med..

[162] Lescroart F., Meilhac S.M., Kubiak J. (2012). Cell lineages, growth and repair of the mouse heart. Mouse Development. Results and Problems in Cell Differentiation.

[163] Niederreither K., Vermot J., Messaddeq N., Schuhbaur B., Chambon P., Dollé P. (2001). Embryonic retinoic acid synthesis is essential for heart morphogenesis in the mouse. Development.

[164] Von Gise A., Zhou B., Honor L.B., Ma Q., Petryk A., Pu W.T. (2011). WT1 regulates epicardial epithelial to mesenchymal transition through β-catenin and retinoic acid signaling pathways. Dev. Biol..

[165] Braitsch C.M., Combs M.D., Quaggin S.E., Yutzey K.E. (2012). Pod1/Tcf21 is regulated by retinoic acid signaling and inhibits differentiation of epicardium-derived cells into smooth muscle in the developing heart. Dev. Biol..

[166] Xavier-Neto J., Shapiro M.D., Houghton L., Rosenthal N. (2000). Sequential programs of retinoic acid synthesis in the myocardial and epicardial layers of the developing avian heart. Dev. Biol..

[167] Pérez-Pomares J.M., Phelps A., Sedmerova M., Carmona R., González-Iriarte M., Muoz-Chápuli R., Wessels A. (2002). Experimental studies on the spatiotemporal expression of WT1 and RALDH2 in the embryonic avian heart: A model for the regulation of myocardial and valvuloseptal development by epicardially derived cells (EPDCs). Dev. Biol..

[168] Guadix J.A., Ruiz-Villalba A., Lettice L., Velecela V., Munoz-Chapuli R., Hastie N.D., Perez-Pomares J.M., Martinez-Estrada O.M. (2011). Wt1 controls retinoic acid signalling in embryonic epicardium through transcriptional activation of Raldh2. Development.

[169] Brade T., Kumar S., Cunningham T.J., Chatzi C., Zhao X., Cavallero S., Li P., Sucov H.M., Ruiz-Lozano P., Duester G. (2011). Retinoic acid stimulates myocardial expansion by induction of hepatic erythropoietin which activates epicardial Igf2. Development.

[170] Stuckmann I., Evans S., Lassar A.B. (2003). Erythropoietin and retinoic acid, secreted from the epicardium, are required for cardiac myocyte proliferation. Dev. Biol..

[171] Kang J.O., Sucov H.M. (2005). Convergent proliferative response and divergent morphogenic pathways induced by epicardial and endocardial signaling in fetal heart development. Mech. Dev..

[172] Lin S.-C., Dolle P., Ryckebusch L., Noseda M., Zaffran S., Schneider M.D., Niederreither K. (2010). Endogenous retinoic acid regulates cardiac progenitor differentiation. Proc. Natl. Acad. Sci. USA.

[173] Merki E., Zamora M., Raya A., Kawakami Y., Wang J., Zhang X., Burch J., Kubalak S.W., Kaliman P., Belmonte J.C.I. (2005). Epicardial retinoid X receptor is required for myocardial growth and coronary artery formation. Proc. Natl. Acad. Sci. USA.

[174] Lavine K.J., Yu K., White A.C., Zhang X., Smith C., Partanen J., Ornitz D.M. (2005). Endocardial and epicardial derived FGF signals regulate myocardial proliferation and differentiation in vivo. Dev. Cell.

[175] Itou J., Oishi I., Kawakami H., Glass T.J., Richter J., Johnson A., Lund T.C., Kawakami Y. (2012). Migration of cardiomyocytes is essential for heart regeneration in zebrafish. Development.

[176] Lepilina A., Coon A.N., Kikuchi K., Holdway J.E., Roberts R.W., Burns C.G., Poss K.D. (2006). A Dynamic Epicardial Injury Response Supports Progenitor Cell Activity during Zebrafish Heart Regeneration. Cell.

[177] Kikuchi K., Holdway J.E., Major R.J., Blum N., Dahn R.D., Begemann G., Poss K.D. (2011). Retinoic acid production by endocardium and epicardium is an injury response essential for zebrafish heart regeneration. Dev. Cell.

